# Organ-Specific Silica Body Morphology and Developmental Silicification in Leaf Blades of *Zingeria biebersteiniana*

**DOI:** 10.3390/plants15213476

**Published:** 2026-11-06

**Authors:** Shahzad Hussain Shah, Kristi Spieleder, Ryan Koeth, Changbin Chen

**Affiliations:** School of Life Sciences, Arizona State University, Tempe, AZ 85287, USA; sshah246@asu.edu (S.H.S.); kspieled@asu.edu (K.S.); ryankoeth3@gmail.com (R.K.)

**Keywords:** phytolith, dry-ash imaging, fluorescence microscopy, *in vitro*-grown, grass anatomy, leaf development, silicon biology, plant opal

## Abstract

Silicon deposition in grasses produces silica bodies with characteristic morphologies and distributions, but our understanding of their organ-specific diversity and developmental silicification remains limited beyond extensively studied crop species. Here, we investigated silica body formation in *Zingeria biebersteiniana*, a diploid grass with 2*n* = 4 chromosomes, by combining organ-level morphological characterization with a post-transplant developmental analysis. Dry-ash preparations were examined by fluorescence microscopy and quantified using digital image analysis. Mature soil-grown plants exhibited organ-associated silica body morphotypes in leaf blades, leaf sheaths, stems, glumes, lemmas, and paleae, with marked differences in morphology, distribution, and epidermal coverage. Plants maintained *in vitro* on Murashige and Skoog (MS) medium without intentionally added Si showed little to no detectable leaf silicification. Following transfer to soil, silica bodies were readily detectable by Day 7 in both Leaf 3, which was fully expanded at transplantation, and the younger, developing Leaf 2. Descriptive analysis showed broad silica-body area distributions that shifted during the post-transplant period. Spatial imaging further revealed interplant heterogeneity: three plants showed more distal and marginal silicification, whereas nine showed relatively uniform blade-wide patterns. These results provide an anatomical and developmental framework for investigating silicon biology in *Z. biebersteiniana* and demonstrate the utility and limitations of fluorescence-based dry-ash imaging for descriptive analysis of silicification in grasses.

## 1. Introduction

Silicon (Si) is one of the most abundant mineral elements in the Earth’s crust and is taken up by plants as soluble monosilicic acid (Si(OH)_4_) from the soil solution [1,2,3]. Although silicon is not considered an essential nutrient for most plants, its accumulation has been associated with numerous beneficial effects, including enhanced mechanical strength, improved tolerance to abiotic stresses such as drought and salinity, and increased resistance to pathogens and herbivores [4,5,6]. The amount of silicon accumulated varies greatly among plant species, with grasses (Poaceae) representing one of the largest groups of silicon accumulators, frequently containing several percent silicon on a dry weight basis [7,8,9]. After uptake by roots, Si is transported through the transpiration stream and polymerizes as amorphous hydrated silica (SiO_2_·nH_2_O) within specific tissues and cell types [10].

In grasses, Si is deposited in specialized epidermal cells to form silica bodies (also referred to as phytoliths, or plant opal), whose morphology and distribution vary among organs, species, and taxonomic groups [11,12,13]. Silica bodies have long attracted attention because of their taxonomic value, exceptional preservation in soils and sediments, and widespread applications in archaeology, paleoecology, and paleobotany [11,14,15,16,17,18,19]. More recently, studies have emphasized their biological significance, demonstrating that silicification is a highly regulated developmental process rather than a simple consequence of silica precipitation during transpiration [20]. Evidence from sorghum and other grasses has shown that silica deposition occurs in living silica cells and is developmentally controlled, suggesting that specialized cellular mechanisms regulate silicification during leaf maturation [21].

Methods for studying silica bodies have evolved substantially with advances in microscopy. Early studies used bright-field microscopy of epidermal preparations to characterize silica body morphology and distribution [22,23,24], while dry-ash preparations allowed silica bodies to be examined *in situ* while preserving their spatial arrangement within plant tissues [11,25]. Scanning electron microscopy subsequently provided higher-resolution characterization of silica body morphology and surface structure [26,27]. More recently, fluorescence microscopy coupled with digital image analysis has enabled rapid visualization and quantitative analysis of silica bodies, facilitating comparative and developmental studies of silicification [28,29,30,31].

Comparative studies have established substantial diversity in silica body morphology among grasses and demonstrated relationships between silica body morphotypes and grass taxonomy [11,18,32,33]. Developmental studies have further shown that silica body morphology, size, and arrangement can change during organ and leaf development [12,27]. Nevertheless, most investigations have focused on economically important grasses or isolated phytoliths for taxonomic and paleoenvironmental applications [27,30,34,35,36]. Relatively little is known about organ-specific silica body morphology and the dynamics of silicification during leaf development in wild grasses. Furthermore, few studies have combined anatomical observations with quantitative analyses of silica body development within the same experimental framework.

*Zingeria biebersteiniana* (syn. *Colpodium biebersteinianum*) is a diploid grass species possessing the lowest known chromosome number among flowering plants (2*n* = 4), making it an attractive system for genetics, genomics, and developmental studies [35,36,37,38,39,40]. As part of our efforts to establish *Z. biebersteiniana* as a model grass species, our laboratory has developed genomic resources, transformation protocols, and imaging approaches that facilitate studies of plant development and cell differentiation [41,42,43]. Characterizing silica body formation in this species provides an important anatomical foundation for future investigations into the genetic regulation of silicification and its relationship to leaf development and environmental adaptation.

In the present study, we employed fluorescence microscopy combined with dry-ash imaging and quantitative image analysis to investigate silica body development in *Z. biebersteiniana* [28]. Specifically, we (1) characterized silica body morphology and distribution in vegetative and reproductive organs, including leaf blades, leaf sheaths, stems, glumes, lemmas, and paleae; (2) examined the developmental establishment of silicification in leaf blades following the transfer of *in vitro*-grown plants from Murashige and Skoog (MS) medium without intentionally added Si to soil; and (3) quantified changes in silica body size distributions during leaf development. Together, these analyses provide an integrated description of organ-specific silica body morphology and developmental silicification in *Z. biebersteiniana* and establish a framework for future studies of silicon biology in this emerging grass model.

## 2. Materials and Methods

This study consisted of two complementary experiments: (1) characterization of silica body morphology in different vegetative and floral organs of mature soil-grown plants, and (2) developmental analysis of silica body deposition in *in vitro*-grown plants following transfer from MS medium without added Si to soil.

### 2.1. Soil-Grown Plants

Wild-type seeds of *Zingeria biebersteiniana* were propagated from germplasm obtained from the USDA Agricultural Research Service (USDA-ARS) Germplasm Resources Information Network (GRIN), W6 station (accession no. 19209), originally collected in Turkey [44]. Seeds were placed on blue blotter paper (Anchor Paper Co., St. Paul, MN, USA) moistened with distilled water or half-strength Murashige and Skoog (MS) basal salt medium (PhytoTech labs, Lenexa, KS, USA) in 100 mm plastic Petri dishes (Thermo Fisher Scientific, Waltham, MA, USA, 20–30 seeds per dish). Plates were sealed with Parafilm, wrapped in aluminum foil, and cold-stratified at 4 °C for 2–3 days.

Following stratification, plates with seeds were transferred to a growth chamber (Conviron Plant Growth Chamber model E8, Pembina, ND, USA) under a 16 h light/8 h dark photoperiod (~100: 99–107 µmol m^−2^ s^−1^) at 22 °C. After germination, seedlings were transplanted into pots containing a soil mixture of 50% OMNI Seed Cover & Planting Compost (OMN71260), 25% sand, and 25% clay. Newly transplanted seedlings were covered with transparent plastic domes to maintain high humidity for 3–4 days before gradual acclimatization.

Plants were subsequently grown in a growth chamber under the same photoperiod and temperature, with a light intensity of ~250 (234–253) µmol m^−2^ s^−1^. A water-soluble fertilizer (Peters Professional 20-20-20; ICL Specialty Fertilizers) was applied at 0.5 g L^−1^ every two weeks. After 2–3 months, mature plants were harvested for silica body characterization in the leaf blade, leaf sheath, stem, glume, lemma, and palea.

### 2.2. Growth of Plants on In Vitro MS Medium Without Intentionally Added Si and Acclimatization

*Zingeria biebersteiniana* seeds were grown *in vitro* on MS medium. Seeds were surface-sterilized in a 1:1 mixture of 70% ethanol (Decon Labs, King of Prussia, PA, USA) and 50% (*w*/*w*) hydrogen peroxide in H_2_O, stabilized (Sigma-Aldrich, St. Louis, MO, USA) for 3 min and rinsed three times with deionized water. Seeds were then dried on sterile filter/blotting paper and plated on medium containing MS basal salt mixture (4 g L^−1^), sucrose (30 g L^−1^; Thermo Fisher Scientific), agar (10 g L^−1^; Sigma-Aldrich), and Plant Preservative Mixture (PPM; 2 mL L^−1^; PhytoTech Labs) [44]. No Si source was intentionally added to the medium. Plates were wrapped in aluminum foil and stratified at 4 °C for 2–3 days before transfer to a growth chamber. After germination, seedlings were transferred to vented plastic culture vessels (PhytoTech Labs) containing the same MS medium. Seedlings were grown *in vitro* for 4–5 weeks, transplanted to soil, and covered with transparent plastic domes to maintain high humidity during initial acclimatization. Photoperiod, light intensity, and temperature were the same as those described for soil-grown plants.

### 2.3. Sample Collection

For the organ-specific morphological study, samples of the leaf blade, leaf sheath, stem, and inflorescence were collected from mature soil-grown plants. Individual floral organs, including the glume, lemma, and palea, were carefully dissected from mature inflorescences prior to silica body analysis.

For the developmental study, *in vitro*-cultured seedlings were transferred from MS medium to soil, and leaf samples were collected at Day 0, Day 7, and Day 14 after transplantation, which is illustrated in Figure 4. At Day 0, approximately 1 cm of tissue was excised from the tip of the third leaf (Leaf 3), representing an expanded leaf at the time of transplantation. At Days 7 and 14, additional sectors were collected from the same Leaf 3 on each plant to monitor the establishment of silica body deposition during acclimatization.

To compare silicification between mature and developing leaves, the second leaf (Leaf 2), which was a young and developing leaf at the time of transplantation, was sampled at Days 7 and 14. Approximately 1 cm of tissue was collected from the leaf tip for developmental analyses.

### 2.4. Dry-Ash Preparation, Fluorescence Microscopy, and Image Analysis

Dry-ash preparations were produced following our previously described fluorescence-based silica-body imaging method [28], with a furnace-based modification to improve sample-processing efficiency. The method removes organic tissue while preserving mineral silica residues in their approximate spatial positions, enabling fluorescence imaging of silica-body morphology and projected distribution. Because cellular organic material is removed, the same preparation cannot be co-labeled for nuclei or intact cell walls using DAPI, SYTOX, or calcofluor; complementary intact-tissue imaging would be required for per-cell normalization.

Leaf samples were placed between two 25 × 75 × 1 mm glass microscope slides (VWR, Radnor, PA, USA) to minimize sample displacement during ashing. The slide assembly was heated in a RapidFire Pro-L Standard Furnace (TableTop Furnace Co., Lake Katrine, NY, USA) at 600 °C for 10 min until the tissues were completely ashed. Compared with our previous hot-plate protocol [11,28], the furnace-based procedure substantially reduced sample preparation time while producing consistent ash preparations suitable for fluorescence imaging. After cooling to room temperature, the upper slide was carefully removed, and a small drop of cedarwood oil (Electron Microscopy Sciences, Hatfield, PA, USA) was applied to the ash preparation. A 22 × 22 mm No. 1 coverslip (VWR, Radnor, PA, USA) was then placed over the sample, and the slide was briefly warmed over a low flame to promote uniform distribution of the mounting medium and minimize trapped air bubbles. The same procedure was used to prepare samples from the leaf blade, leaf sheath, stem, glume, lemma, and palea collected from mature soil-grown plants.

Prepared slides were examined using a Nikon Eclipse fluorescence microscope (Nikon Instruments Inc., Melville, NY, USA) equipped with a GFP filter set (Excitation: 470/40; Dichroic: 495; Emission: 525/50) and an Iris 15 camera (Teledyne Photometrics, Tucson, AZ, USA) or a Nikon DS-Ri2 camera. Silica bodies exhibited strong green autofluorescence against a dark background, enabling their visualization without additional staining. Images were acquired at magnifications ranging from 40× to 800×, with 200× used for quantitative measurements of silica body size. For each field of view, Z-stack images were captured using NIS-Elements Advanced Research (Ar) software version 5.30.02 (Nikon Instruments Inc. Melville, NY, USA) and rendered into a single focused image using Helicon Focus 8 (Helicon Soft Ltd., Kharkiv, Ukraine).

Image calibration was performed in NIS-Elements using microscope calibration data, and measurements were analyzed in ImageJ version 1.51w (National Institutes of Health, Bethesda, MD, USA). Silica-body projected area was quantified using Adobe Photoshop 2025 Release 26.11.7 (Adobe Inc., San Jose, CA, USA) following the previously described workflow [28]. Image contrast was adjusted to distinguish the strong silica-body autofluorescence from background, and fluorescent objects were selected using the Magic Wand tool. The Magic Wand tool was used with a tolerance setting of 40, which was applied consistently to all images included in the quantitative analysis. Sample identity was known during image analysis. All images included in a given comparison were processed using the same general calibrated workflow by the study team. Future applications should record fixed segmentation parameters prospectively and validate automated selections against manual or independent image-analysis measurements.

To quantify silica-body coverage among organs, the leaf blade, leaf sheath, stem, glume, lemma, and palea were treated as separate samples, and multiple images were collected from each organ for morphological characterization. For quantitative analysis, one representative field of view from each of five independent plants was analyzed (n = 5 biological replicates per organ). The representative field was selected from the central visible region of each organ section where the tissue was intact, clearly focused, and free from damage, folding, or other preparation artifacts. All analyzed fields were acquired at the same magnification and processed using the same calibrated area-based workflow. Silica-body coverage was calculated for each field as the total pixel area occupied by segmented silica bodies divided by the total analyzed image area × 100. The five plant-level coverage measurements were then used to calculate the mean and standard deviation for each organ. Coverage was expressed as percentage epidermal area rather than normalized to epidermal cell number because clearly identifiable cell boundaries are not retained after dry-ash preparation.

### 2.5. Statistical Analysis

For organ-level silica-body coverage, measurements from five independent plants were treated as biological replicates (n = 5 per organ) and summarized as mean ± standard deviation (SD). Because only one retained field per plant was included for the reported coverage dataset and the small sample size limits robust assessment of distributional assumptions, organ-level coverage is presented descriptively without inferential significance testing. For the developmental experiment, 12 independent plants were followed through the post-transplant sampling period, and the mature soil-grown control consisted of five independent plants. Multiple silica bodies were measured within each sampled leaf sector. Silica-body area distributions are summarized using mean, SD, median, and range and visualized using violin plots. Individual silica bodies are nested observations and were not treated as independent biological replicates. Plant-level summary values needed for a rigorous repeated-measures or mixed-effects comparison were not preserved in the current dataset; therefore, no inferential claims are made about differences among post-transplant groups. Future experiments will retain plant-level measurements and use repeated-measures or mixed-effects models as appropriate.

## 3. Results

### 3.1. Organ-Specific Distribution and Morphology of Silica Bodies in Zingeria biebersteiniana

**Figure 1:**
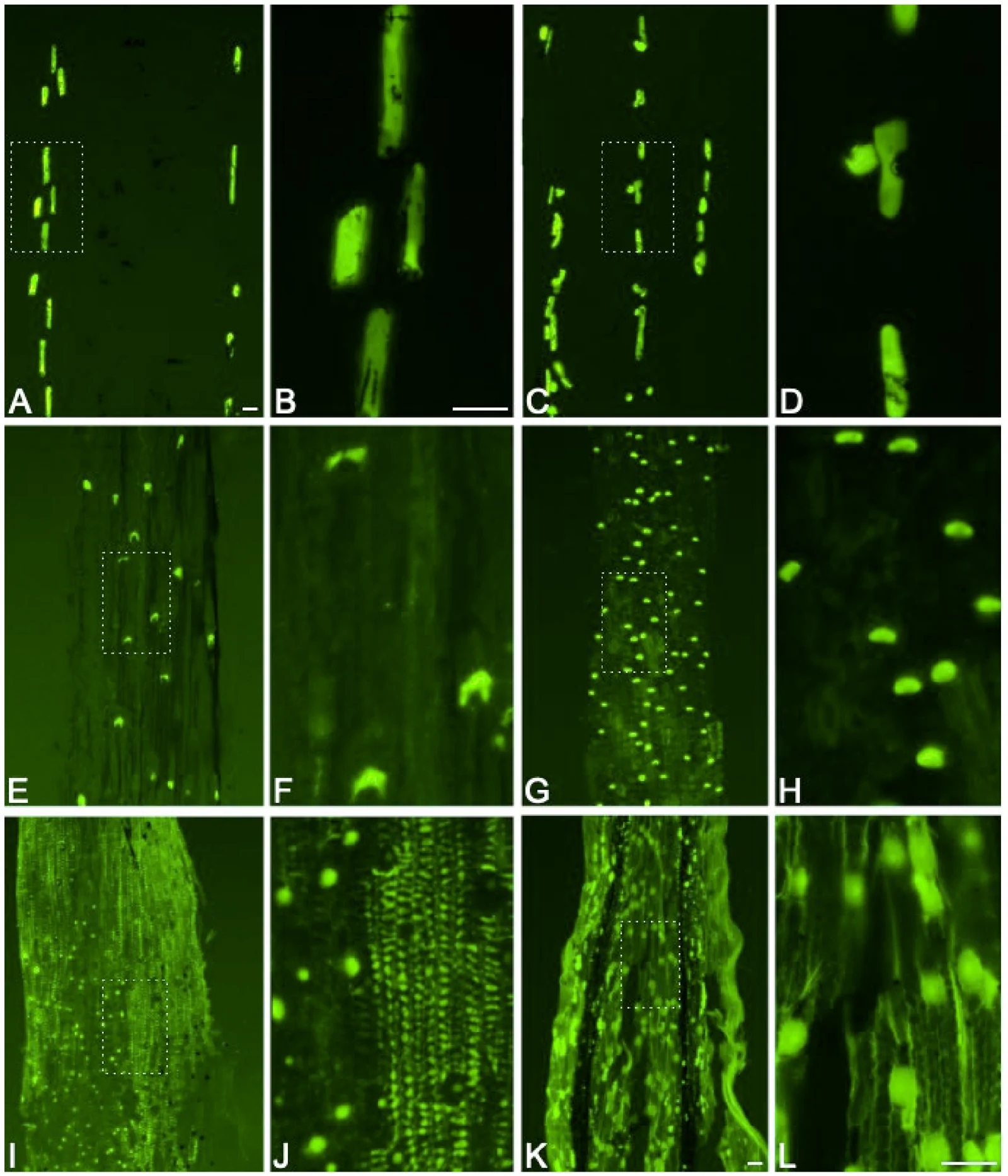
Silica body morphotypes and distribution patterns in different organs of *Zingeria biebersteiniana*. (**A**,**B**) Leaf blade; (**C**,**D**) leaf sheath; (**E**,**F**) stem; (**G**,**H**) glume; (**I**,**J**) lemma; and (**K**,**L**) palea. Panels (**B**), (**D**), (**F**), (**H**), (**J**), and (**L**) (same magnification) are higher-magnification views of the regions outlined by dotted lines in panels (**A**), (**C**), (**E**), (**G**), (**I**), and (**K**) (same magnification), respectively. All scale bars = 10 µm.

Silica bodies were examined in six organs of *Zingeria biebersteiniana*: the leaf blade, leaf sheath, stem, glume, lemma, and palea (Figure 1; Table 1). Silica body morphology, distribution, and epidermal coverage differed among the organs examined. In the leaf blade, the predominant silica bodies were elongate, with less frequent oblong forms. They occurred in well-defined longitudinal files associated with the leaf epidermis (Figure 1A,B). Silica bodies in the leaf sheath were predominantly oblong and were also arranged in longitudinal files, although their distribution appeared less regular than that observed in the leaf blade (Figure 1C,D).

The stem contained fewer silica bodies than the leaf blade and leaf sheath. These were predominantly reniform and occurred more sparsely within the epidermis, primarily in short silica cells (Figure 1E,F). Distinct silica body morphotypes and distribution patterns were also observed in the glume, lemma, and palea. The glume contained predominantly reniform silica bodies, together with ellipsoidal forms, primarily in short silica cells (Figure 1G,H). The lemma and palea exhibited regional differences in silicification. In both organs, the marginal regions consisted of densely arranged small epidermal cells, most of which contained correspondingly small silica bodies. In the broader central regions, silica bodies were less densely distributed and occurred predominantly in short silica cells. In the lemma, numerous small, predominantly reniform silica bodies occurred in the densely cellular marginal regions, whereas larger circular silica bodies associated with short silica cells occurred in the central region. The palea showed greater morphological heterogeneity. In addition to numerous small reniform silica bodies in the marginal regions, elongate polylobate to irregularly wavy silica bodies occurred along portions of the margins. The central region of the palea was characterized primarily by barrel-shaped silica bodies associated with short silica cells (Figure 1K,L). These organ-associated morphotypes are summarized in Table 1.

### 3.2. Quantitative Analysis of Silica-Body Size and Epidermal Coverage

**Figure 2:**
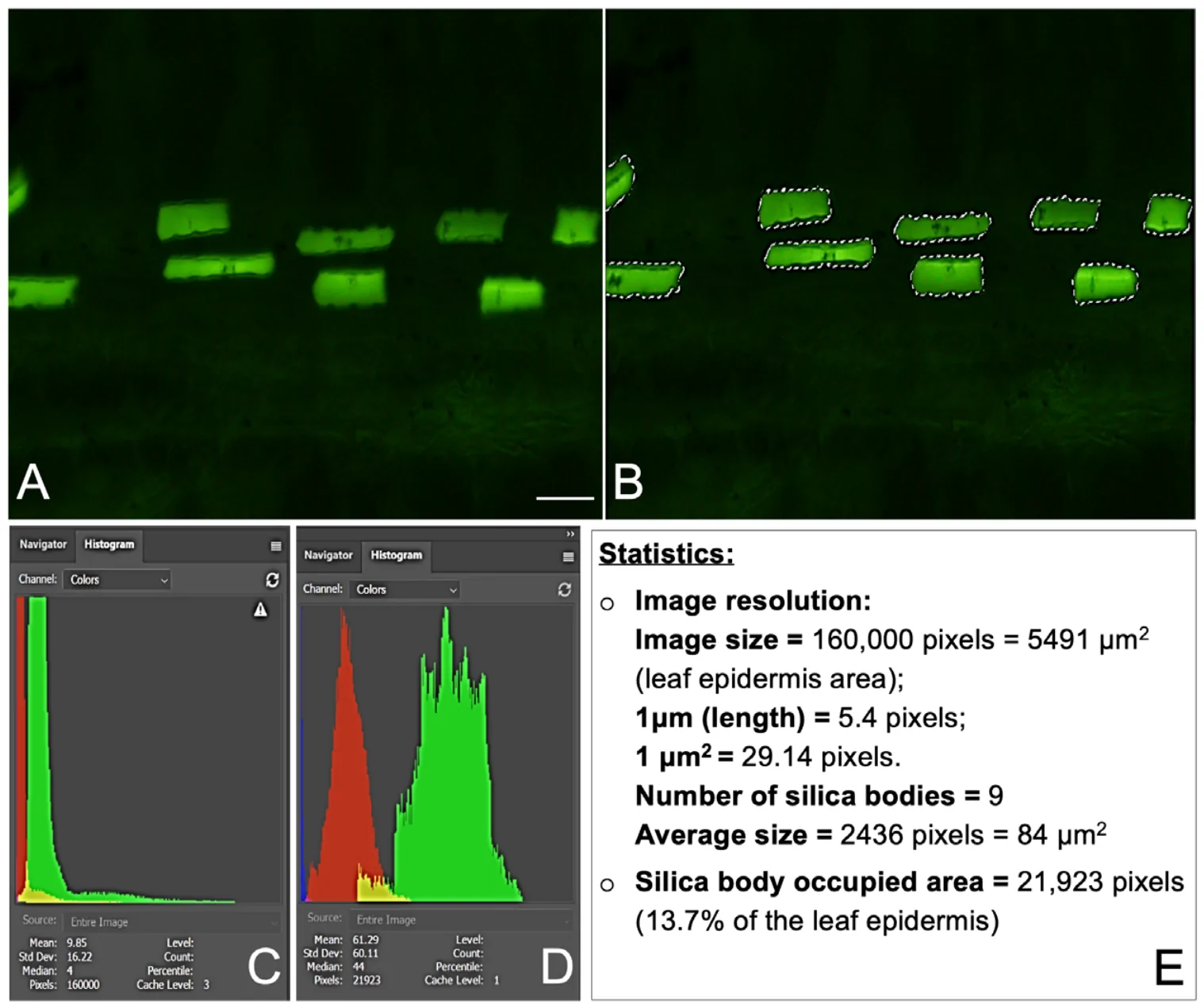
Workflow for quantifying silica body size and coverage in a representative *Zingeria biebersteiniana* dry-ash image using Adobe Photoshop 2025. (**A**) Dry-ash leaf preparation; (**B**) selection of silica bodies using the Magic Wand tool; (**C**) pixel measurements for the entire image; (**D**) pixel measurements for the selected silica bodies; and (**E**) calculation of silica body number, mean area, and percentage epidermal coverage. Scale bar = 10 µm.

Dry-ash preparations examined by fluorescence microscopy enabled silica bodies to be readily distinguished by their strong green autofluorescence against a dark background (Figure 2A) [28]. Individual silica bodies could then be segmented and quantified based on calibrated image area (Figure 2B–E). In the representative mature leaf-blade image shown in Figure 2, the analyzed epidermal area was 5491 µm^2^ and contained nine silica bodies. The silica bodies collectively occupied approximately 13.7% of the analyzed area, with a mean individual silica body area of approximately 84 µm^2^. These measurements illustrate the quantitative approach used subsequently to characterize silica body size distributions during leaf development [28].

**Figure 3:**
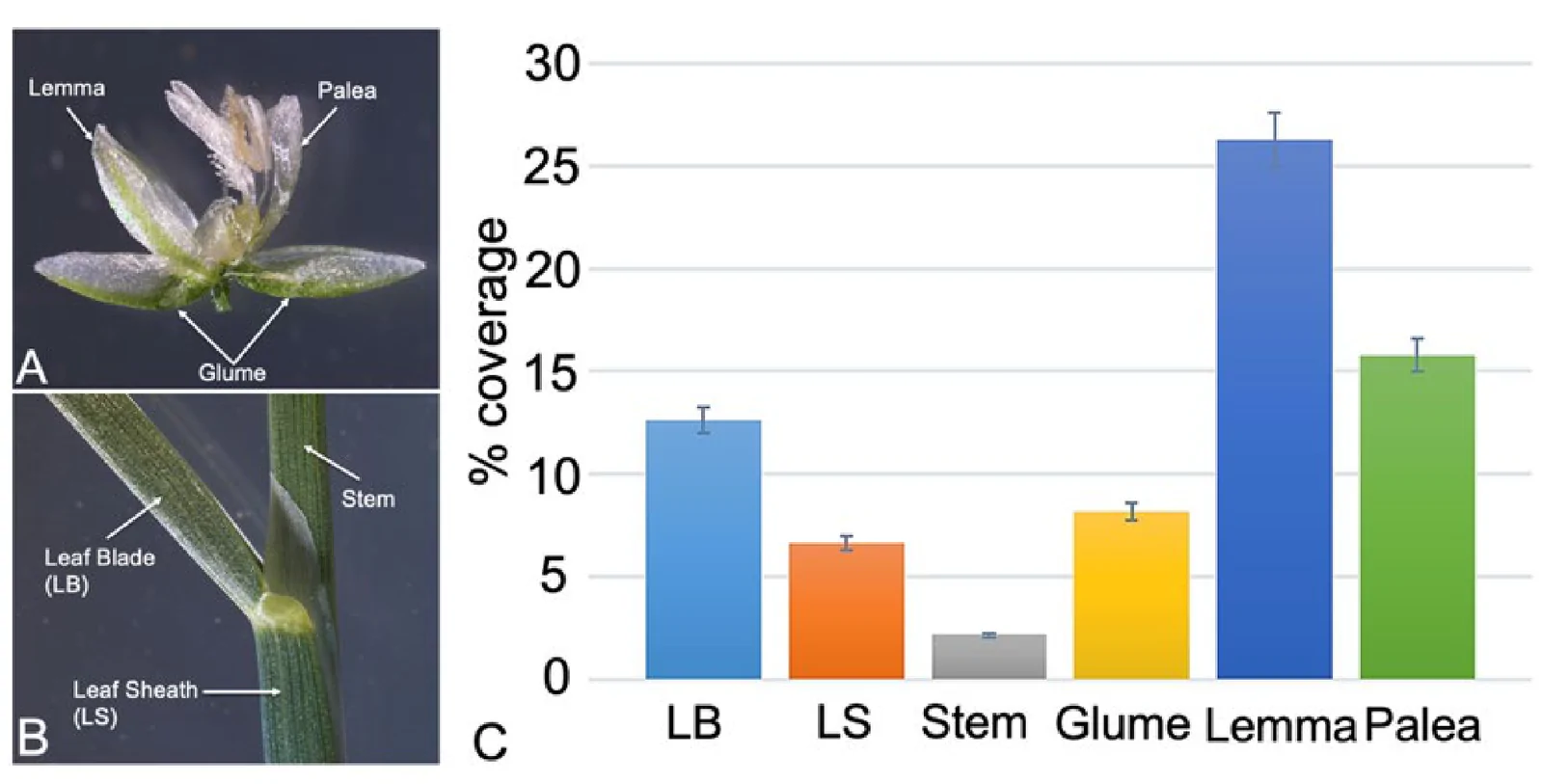
Silica body coverage in different organs of *Z. biebersteiniana*. (**A**) Representative floral organs, including glume, lemma, and palea. (**B**) Representative vegetative organs, including leaf blade (LB), leaf sheath (LS), and stem. (**C**) Percentage of the analyzed epidermal area occupied by silica bodies in the six organs. Multiple images were collected from each organ for morphological examination; one representative field from each of five independent plants was used for quantitative coverage analysis. Values are presented as mean ± SD (n = 5 biological replicates per organ).

For organ-level comparisons, samples were examined from five independent plants. Multiple microscopic images were acquired from each organ to document silica body morphology and distribution. For quantitative analysis of silica body coverage, one representative field from each plant was selected and analyzed using the same magnification and calibrated image-analysis procedure, resulting in five independent measurements per organ (n = 5). Silica body coverage was calculated for each image as the percentage of the analyzed epidermal area occupied by segmented silica bodies, and mean coverage was calculated across the five biological replicates for each organ (Figure 3). Silica bodies in the leaf blade and leaf sheath occurred in epidermal cells with a broader range of sizes and shapes, whereas those in the stem, glume, and central regions of the lemma and palea were predominantly associated with short silica cells.

### 3.3. Silicification Following Transfer of In Vitro-Grown Plants to Soil

**Figure 4:**
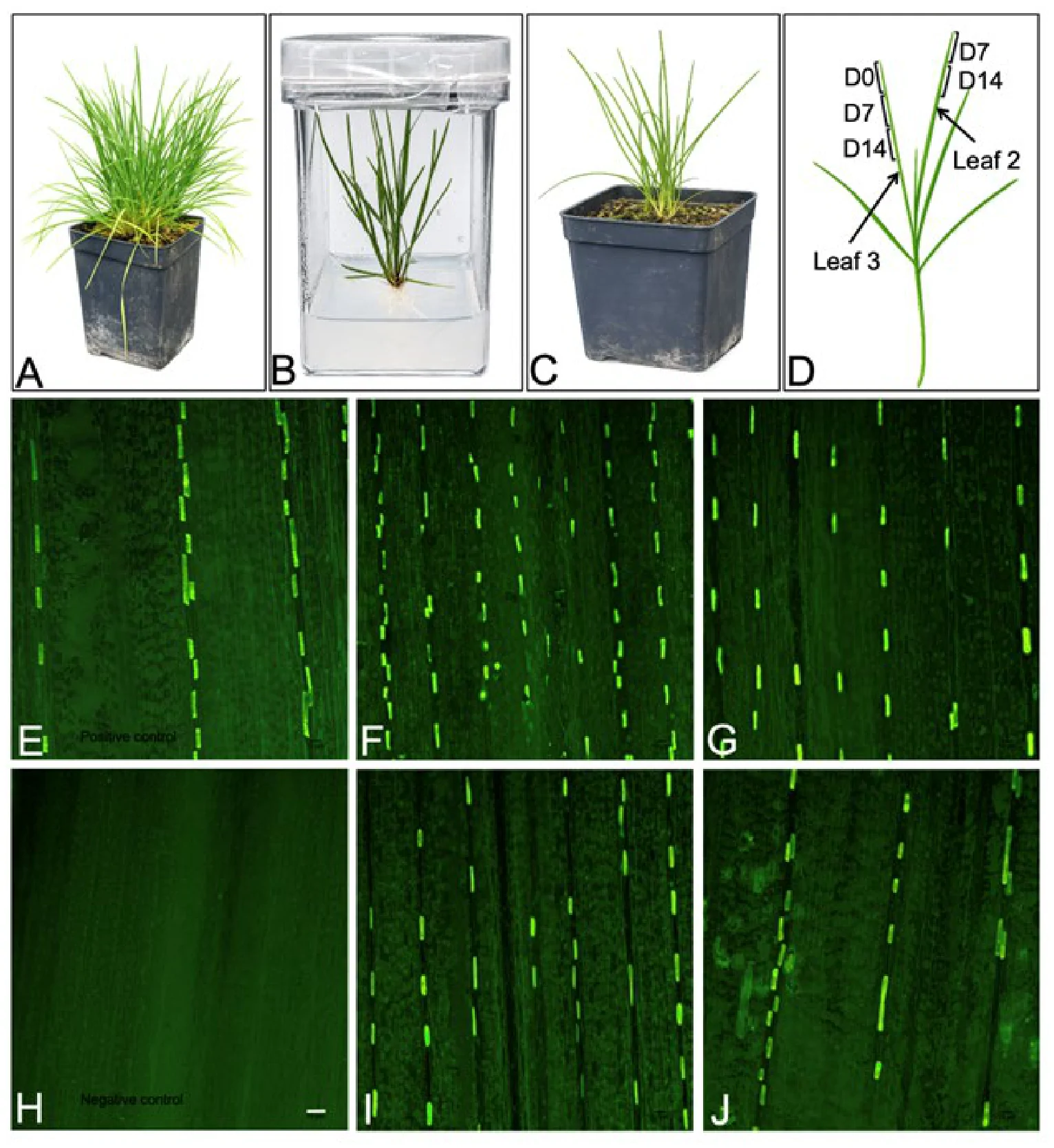
Silica body deposition in leaves of *Zingeria biebersteiniana*. (**A**) Soil-grown plant; (**B**) *in vitro*-grown on MS medium without added Si; (**C**) plant after transfer from MS medium to soil; and (**D**) sampling scheme for the developmental experiment. (**E**) Mature soil-grown leaf (positive control); (**F**,**G**) Leaf 2 at Days 7 and 14 after transfer to soil; (**H**) Leaf 3 at Day 0 (negative control); and (**I**,**J**) Leaf 3 at Days 7 and 14 after transfer to soil. Panels (**E**–**J**) are shown at the same magnification. Scale bar = 10 µm.

Plants maintained on MS medium in plastic culture vessels showed little detectable silicification in the leaf blade compared with mature soil-grown plants (Figure 4). At the time of transfer to soil (Day 0), Leaf 3, which was fully expanded at transplantation, contained essentially no detectable silica bodies and therefore served as the negative control for the developmental experiment. In contrast, mature leaves from soil-grown plants contained abundant, fluorescent silica bodies and served as the positive control.

Following transfer of *in vitro*-grown plants to soil, silica bodies became readily detectable within 7 days. In Leaf 3, which was present at transplantation and served as the Day 0 negative control, numerous silica bodies were observed at Day 7 and remained abundant at Day 14. Silicification was also evident in the younger Leaf 2 at Days 7 and 14, demonstrating silica deposition in both pre-existing mature leaves and younger developing leaves following transfer to soil (Figure 4).

Although both leaves underwent substantial silicification following transplantation, their developmental histories differed. Leaf 3 was fully developed at Day 0 under *in vitro* conditions without added Si and acquired silica bodies after transfer to soil, whereas Leaf 2 was still developing at transplantation and continued its development during the post-transplant period. Together, these observations demonstrate the establishment of silicification in both the fully expanded Leaf 3 and the developing Leaf 2 by Day 7 after transfer from MS medium to soil.

### 3.4. Silica-Body Size Distributions During Post-Transplant Development

**Figure 5:**
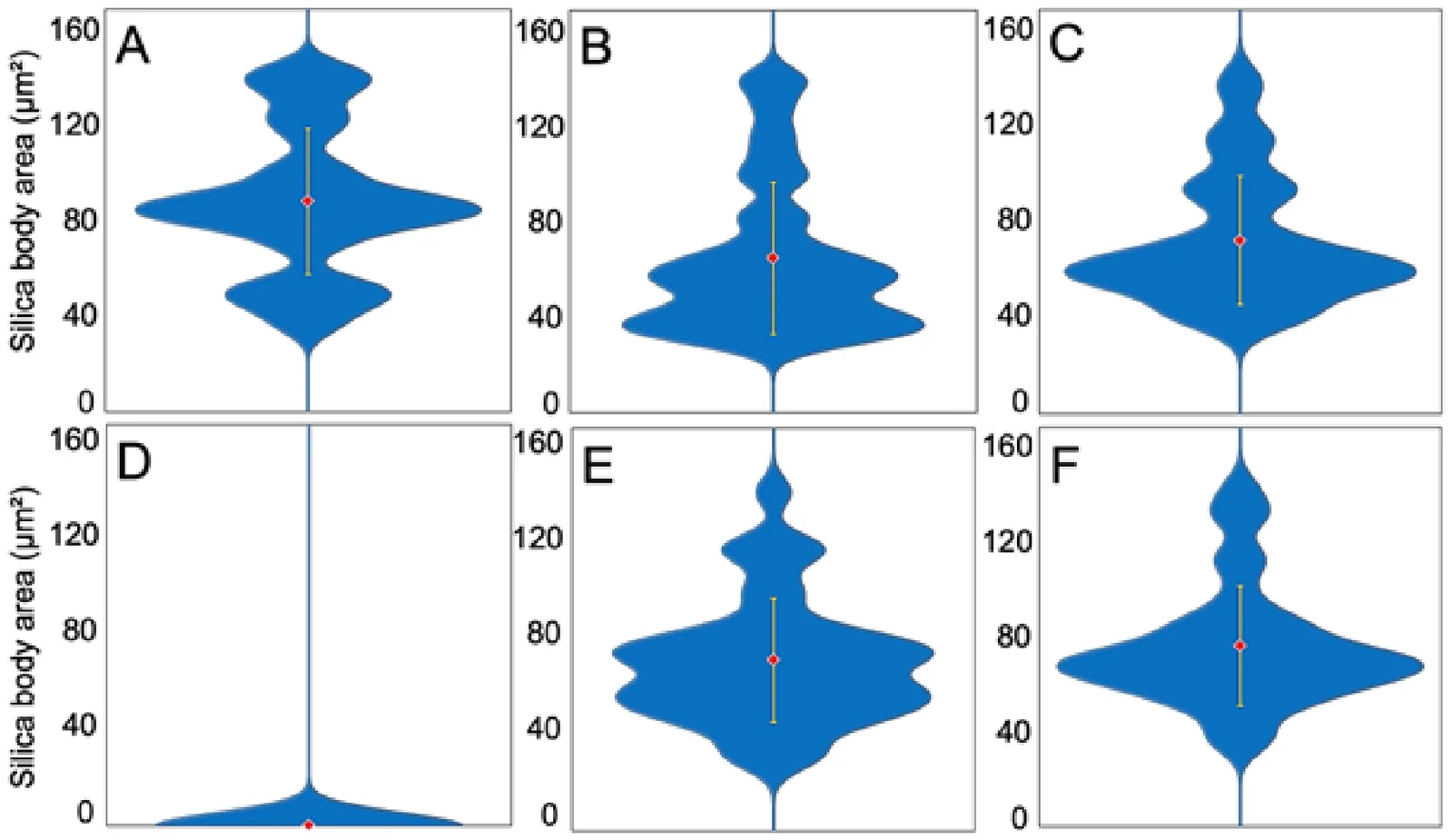
Violin plots showing silica body area distributions in *Z. biebersteiniana*. (**A**) Mature soil-grown leaves (positive control; 5 plants, 259 silica bodies); (**B**,**C**) Leaf 2 at Days 7 and 14 after transfer to soil (12 plants; 376 and 391 silica bodies, respectively); (**D**) Leaf 3 at Day 0 (negative control; no measurable silica bodies); and (**E**,**F**) Leaf 3 at Days 7 and 14 (12 plants; 381 and 386 silica bodies, respectively). Violin plots show pooled descriptive distributions of individual silica-body area measurements (µm^2^). Red diamonds indicate means, and yellow error bars indicate standard deviations.

Descriptive analysis revealed variation in silica-body size distributions between mature soil-grown controls and leaves sampled following transplantation (Figure 5; Table 2). A total of 259 silica bodies were measured from mature soil-grown control plants, with a mean area of 88.9 ± 30.8 µm^2^, a median of 87 µm^2^, and a range of 36–146 µm^2^.

In Leaf 2, which was still developing at the time of transplantation, 376 silica bodies were measured at Day 7 and 391 at Day 14. Mean silica body area increased from 65.3 ± 32.2 µm^2^ at Day 7 to 72.9 ± 27.1 µm^2^ at Day 14, while the median increased from 58 to 64 µm^2^. Silica body sizes ranged from 29–143 µm^2^ at Day 7 and 37–143 µm^2^ at Day 14.

No measurable silica bodies were detected in the fully expanded Leaf 3 at Day 0. Following transfer to soil, however, 381 silica bodies were measured at Day 7 and 386 at Day 14. Mean silica body area increased from 72.2 ± 26.2 µm^2^ at Day 7 to 77.0 ± 25.6 µm^2^ at Day 14, with corresponding medians of 69 and 71 µm^2^. Silica body sizes ranged from 31–144 µm^2^ at Day 7 and 34–146 µm^2^ at Day 14.

The violin plots revealed broad pooled silica-body area distributions, with multiple apparent peaks in several sample groups (Figure 5). These plots are descriptive visualizations of individual silica-body measurements nested within plants and do not represent independent biological replicates or tests of statistical significance. The observed distributions in Leaf 2 and Leaf 3 shifted between Days 7 and 14, but the available data do not distinguish enlargement of existing silica bodies from the appearance of newly silicified bodies or differences in sampling position.

### 3.5. Spatial Pattern of Silica-Body Deposition in Leaf Blades

**Figure 6:**
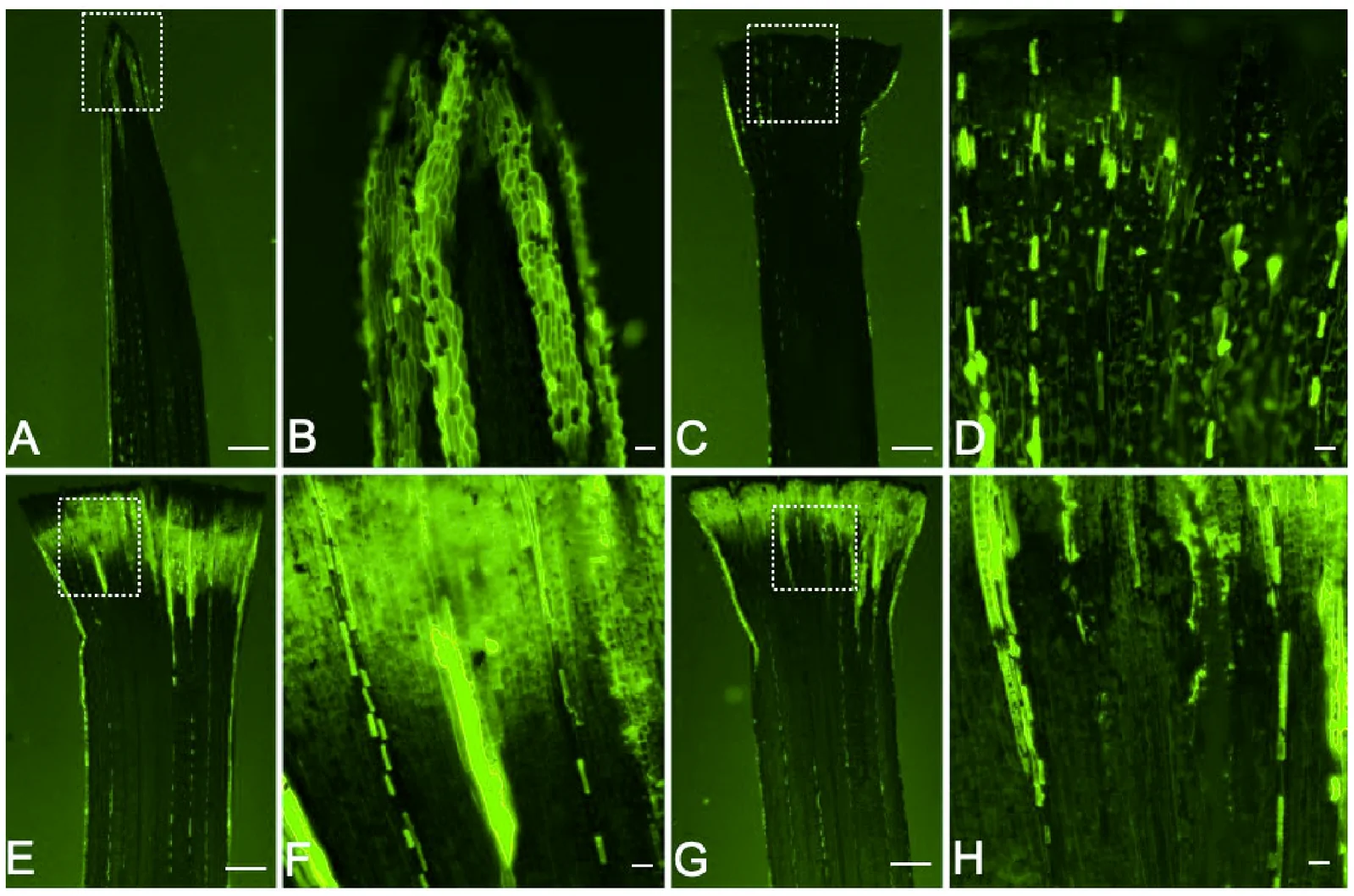
Spatial patterns of silica-body deposition in leaf blades during post-transplant silicification. Samples were collected at 7 and 14 days after transfer from MS medium to soil, and silicification was examined in Leaf 2 (L2) and Leaf 3 (L3). (**A**,**C**) L2 at Days 7 and 14, respectively; (**B**,**D**) higher-magnification views of the boxed regions in (**A**) and (**C**), respectively; (**E**,**G**) L3 at Days 7 and 14, respectively; and (**F**,**H**) higher-magnification views of the boxed regions in (**E**) and (**G**), respectively. Panels (**A**,**C**,**E**,**G**) are shown at the same magnification (scale bar = 100 µm); panels (**B**,**D**,**F**,**H**) are shown at the same magnification (scale bar = 10 µm).

Silica-body deposition showed qualitative spatial variation within leaf blades following transfer of *in vitro*-grown plants to soil (Figure 6). In three plants, visual examination indicated greater silica-body abundance in distal and marginal portions of the blade than in central regions. At later sampling times, silica bodies were observed across a larger portion of the blade, including central regions. Because predefined leaf regions were not quantified, these observations are presented as descriptive spatial patterns rather than as a statistically tested gradient.

This pattern was not observed consistently among plants. Leaves from nine plants appeared relatively uniform across the blade (Supplementary Figure S1). Variation in silica-body size was nevertheless evident within these leaves, with smaller and larger bodies occurring together.

Together, these observations indicate interplant heterogeneity in the visible spatial pattern of silicification. Three plants showed a distal/marginal pattern by qualitative inspection, whereas nine showed a relatively uniform pattern. Quantitative regional sampling will be required to test these apparent spatial differences.

## 4. Discussion

### 4.1. Organ-Specific Diversity of Silica Bodies in Zingeria biebersteiniana

Silica bodies in *Z. biebersteiniana* showed organ-associated differences in morphology, distribution, and epidermal coverage (Figure 1 and Figure 3). Leaf blades and leaf sheaths were dominated by elongate or oblong forms arranged in longitudinal files, whereas stems and glumes contained predominantly reniform silica bodies. Floral bracts showed additional regional differentiation: the lemma contained larger circular silica bodies in its central region and numerous smaller forms near the margins, while the palea combined barrel-shaped central silica bodies with several morphotypes in marginal regions. These observations show that the morphology and spatial pattern of the silica deposits reflect the morphology, position, and differentiation state of the epidermal cells in which deposition occurs. Silicification remains a silica-deposition process, but the resulting phytolith morphotypes are constrained by organ-specific tissue architecture and developmental context. The diversity observed within a single species also cautions against interpreting isolated phytolith morphotypes without considering organ of origin and developmental context.

The organ-specific patterns observed here are consistent with earlier comparative and developmental studies demonstrating that silica body morphology can vary among grass organs and during tissue maturation [12,20,27,37,38]. In *Z. biebersteiniana*, the coexistence of multiple morphotypes within floral structures is particularly notable because floral organs are frequently used in taxonomic and archaeobotanical interpretations [38]. Future comparisons among Zingeria species and related genera could determine which features are stable taxonomic characters and which primarily reflect organ-specific development.

Beyond their value as morphological markers, silica deposits can contribute to mechanical reinforcement and can alter leaf-surface properties relevant to water relations and interactions with herbivores and pathogens. These functions are context-dependent and were not tested in the present study. Accordingly, the organ-specific patterns described here should be viewed as an anatomical foundation for future functional experiments rather than evidence for a particular physiological role in *Z. biebersteiniana.*

### 4.2. Establishment of Silicification After Transfer from In Vitro Culture to Soil

A notable finding of the developmental experiment was that silicification occurred not only in developing leaves but also in leaves that were already fully expanded at the time of transplantation. Leaf 3 was fully expanded and morphologically mature when plants were removed from *in vitro* culture on MS medium without added Si, yet essentially no silica bodies were detectable at Day 0. Following transfer to soil, abundant silica bodies were observed in Leaf 3 within 7 days. In contrast, Leaf 2 was still developing at transplantation and continued its development while undergoing silicification during the post-transplant period (Figure 4 and Figure 5).

Plants acquire Si primarily from the soil solution as soluble silicic acid [Si(OH)_4_], which is taken up with water, transported to aboveground organs, and deposited as amorphous silica in specific cells and tissues [39]. In grasses, the extent and pattern of silicification can be influenced by Si availability, transpiration, tissue age, cell type, and regulation of Si transport [6,39,41,45]. Thus, the appearance of silica bodies after transplantation was expected if the new growth environment provided plant-available Si; however, this experiment did not quantify Si in the MS medium, soil, irrigation water, or plant tissues and did not isolate Si availability from other changes associated with transplantation. The principal result is therefore that a fully expanded leaf retained the capacity to become silicified after transplantation, not that soil Si alone caused the response.

Although the appearance of silica bodies by Day 7 is consistent with access to plant-available Si after transplantation, Si concentrations in the growth media, irrigation water, soil, and plant tissues were not quantified, and transplantation simultaneously altered rooting environment, humidity, nutrient availability, transpiration, and other physiological conditions. Differences in root system size or architecture, root activity, transpiration, and biomass among plants could also influence Si uptake and delivery to leaves. Consequently, the observed response cannot be attributed solely to Si availability. Controlled experiments using defined Si concentrations, matched plant ages, and direct measurements of tissue Si will be required to separate Si-supply effects from transplant-associated physiological changes and to determine the timing and extent of silicification.

### 4.3. Developmental and Spatial Heterogeneity of Silicification in Leaf Blades

The violin plots revealed broad descriptive distributions of silica-body area, with multiple apparent peaks in several sample groups (Figure 5). Mean area was 65.3 µm^2^ in Leaf 2 at Day 7 and 72.9 µm^2^ at Day 14, and 72.2 and 77.0 µm^2^ in Leaf 3 at Days 7 and 14, respectively, compared with 88.9 µm^2^ in mature soil-grown controls. These values are descriptive and were not used to infer statistically significant differences among groups. Because individual silica bodies were not followed through time, the shifts in pooled size distributions cannot be interpreted as direct enlargement of pre-existing silica bodies; they could also reflect formation of new silica bodies, changes in the proportions of developmental classes, or differences in sampled leaf regions. In addition, the mature soil-grown controls were older than the post-transplant plants, so age-normalized comparisons are not possible from the available dataset. Future experiments should use age-matched plants and plant-level longitudinal or mixed-effects analyses.

The broad range of silica-body sizes may reflect heterogeneity among silicified epidermal cells, differences in cell dimensions and developmental stage, and the coexistence of newly silicified and more mature deposits within a leaf. Earlier developmental studies in grasses documented changes in silica-body dimensions and morphology during tissue maturation [12,20,27,40]. The present data extend those observations by documenting substantial within-leaf heterogeneity after transplantation, but they do not resolve whether individual silica bodies enlarge after deposition.

Heterogeneity was also evident in the spatial distribution of silica bodies within leaf blades. In three of the 12 plants examined, visual inspection showed greater silica-body abundance near the distal tip and margins, whereas leaves from the other nine plants appeared relatively uniform at comparable sampling times (Figure 6 and Figure S1). Because basal, middle, distal, and marginal regions were not sampled using a predefined quantitative grid, this classification should be regarded as qualitative rather than as a statistically tested spatial gradient. Variation among plants could reflect differences in leaf developmental state, transpiration, local epidermal differentiation, root architecture or biomass, and resulting differences in Si uptake and transport. Future studies should quantify coverage or density in predefined leaf regions and relate those measurements to plant-level Si status and root traits.

### 4.4. Methodological Implications

The fluorescence-based dry-ash approach was useful because it preserved the approximate spatial arrangement of mineral silica residues while allowing rapid visualization and digital quantification over relatively large areas. The furnace-based modification shortened sample preparation relative to the earlier hot-plate procedure [28,31]. The method is therefore well suited to descriptive comparisons of silica-body morphology, projected area, and spatial distribution. Its limitations are equally important: dry ashing removes nuclei and cell walls, preventing same-preparation cellular co-labeling or normalization per intact epidermal cell, and the two-dimensional projected-area metric does not estimate silica volume. The approach complements, rather than replaces, intact-tissue microscopy and elemental or molecular methods. SEM-EDS can characterize local elemental composition, ICP-OES can quantify tissue Si, and molecular studies of Si transporters can address mechanisms of uptake and transport [1,41,42,43,45,46,47].

## 5. Conclusions

This study establishes an organ-level morphological framework for silicification in *Zingeria biebersteiniana* and documents the post-transplant appearance of silica bodies in both a developing leaf and a leaf that was already fully expanded at transplantation. The principal new finding is that the morphological maturity of the leaf did not preclude subsequent silicification under the post-transplant conditions examined. The study also reveals substantial heterogeneity in silica-body morphology, projected area, and spatial distribution, while highlighting important limits of the present design: Si supply and tissue Si were not quantified, plant ages differed between mature controls and post-transplant groups, spatial patterns were assessed qualitatively, and pooled silica-body measurements do not provide plant-level inferential tests. Accordingly, the data support descriptive developmental associations rather than a causal mechanism of Si uptake or deposition. Future work should combine defined Si treatments, age-matched controls, plant-level longitudinal statistics, regional leaf sampling, elemental quantification, and intact-tissue/cellular imaging to determine how Si availability, root and transpiration traits, epidermal development, and transport processes regulate silicification. The fluorescence-based dry-ash approach provides an efficient complementary platform for such studies by preserving mineral morphology and spatial pattern for high-throughput descriptive imaging.

## Figures and Tables

**Table 1:** Organ-specific morphotypes of silica bodies in *Z. biebersteiniana*.

Plant Parts	Main Silica Body Type		Other Silica Body Types		
Leaf Blade	elongate	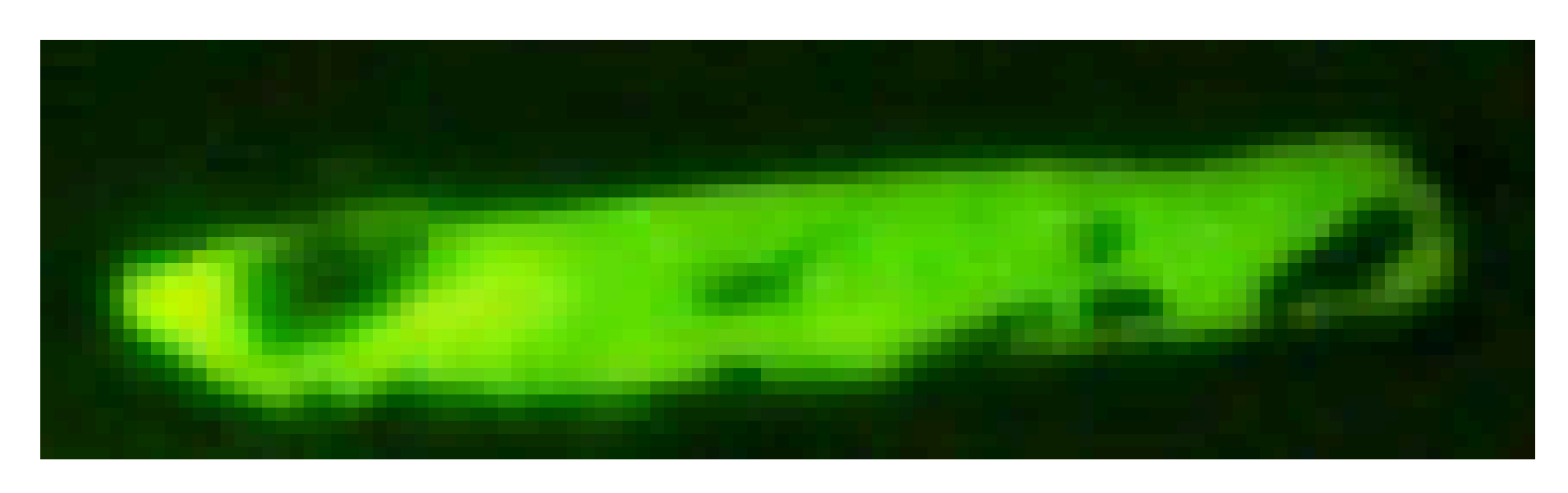	oblong	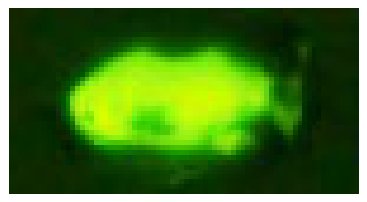	
Leaf Sheath	oblong	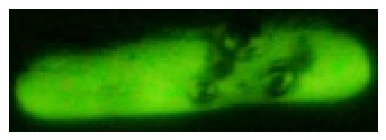			
Stem	reniform	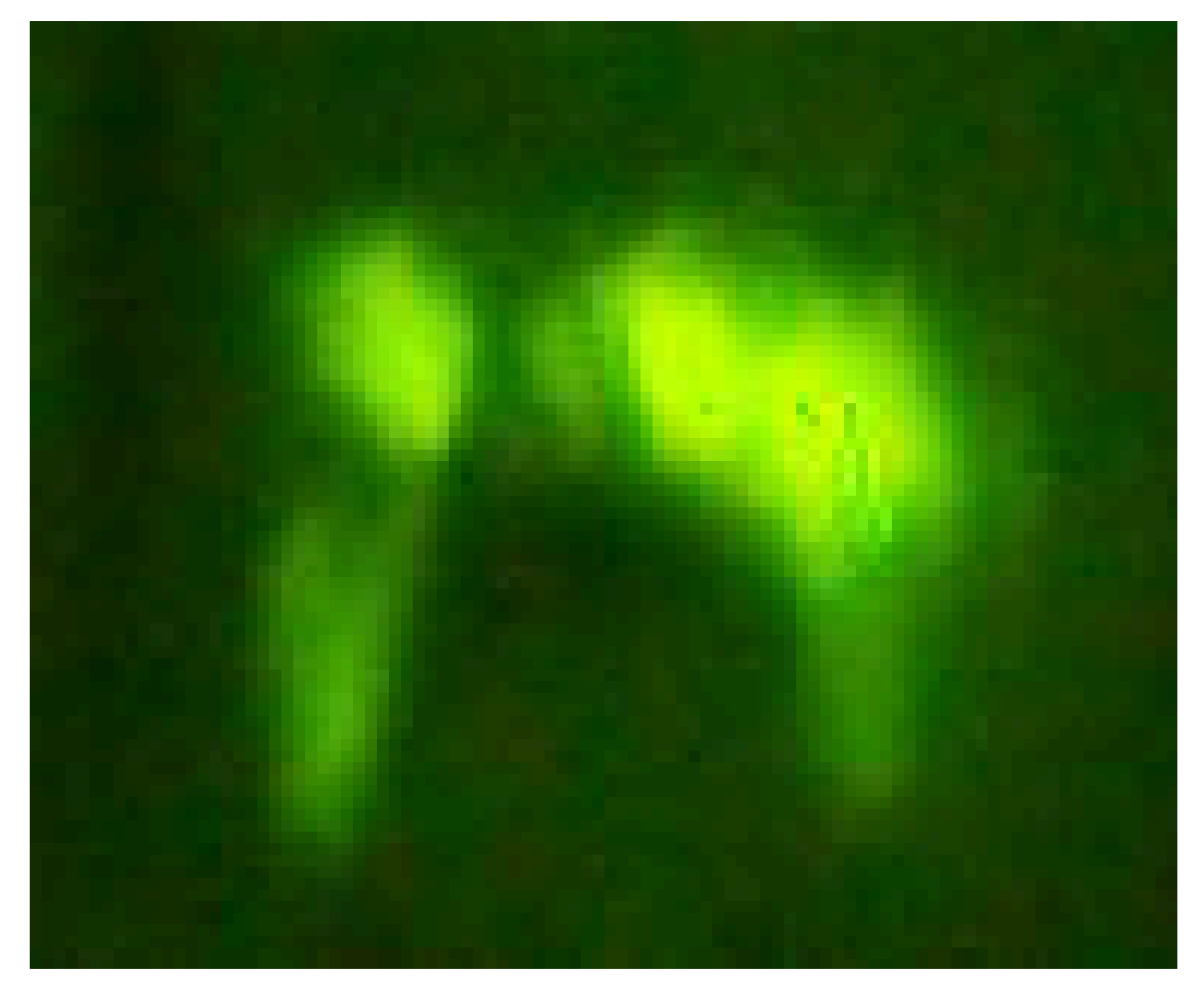			
Glume	reniform	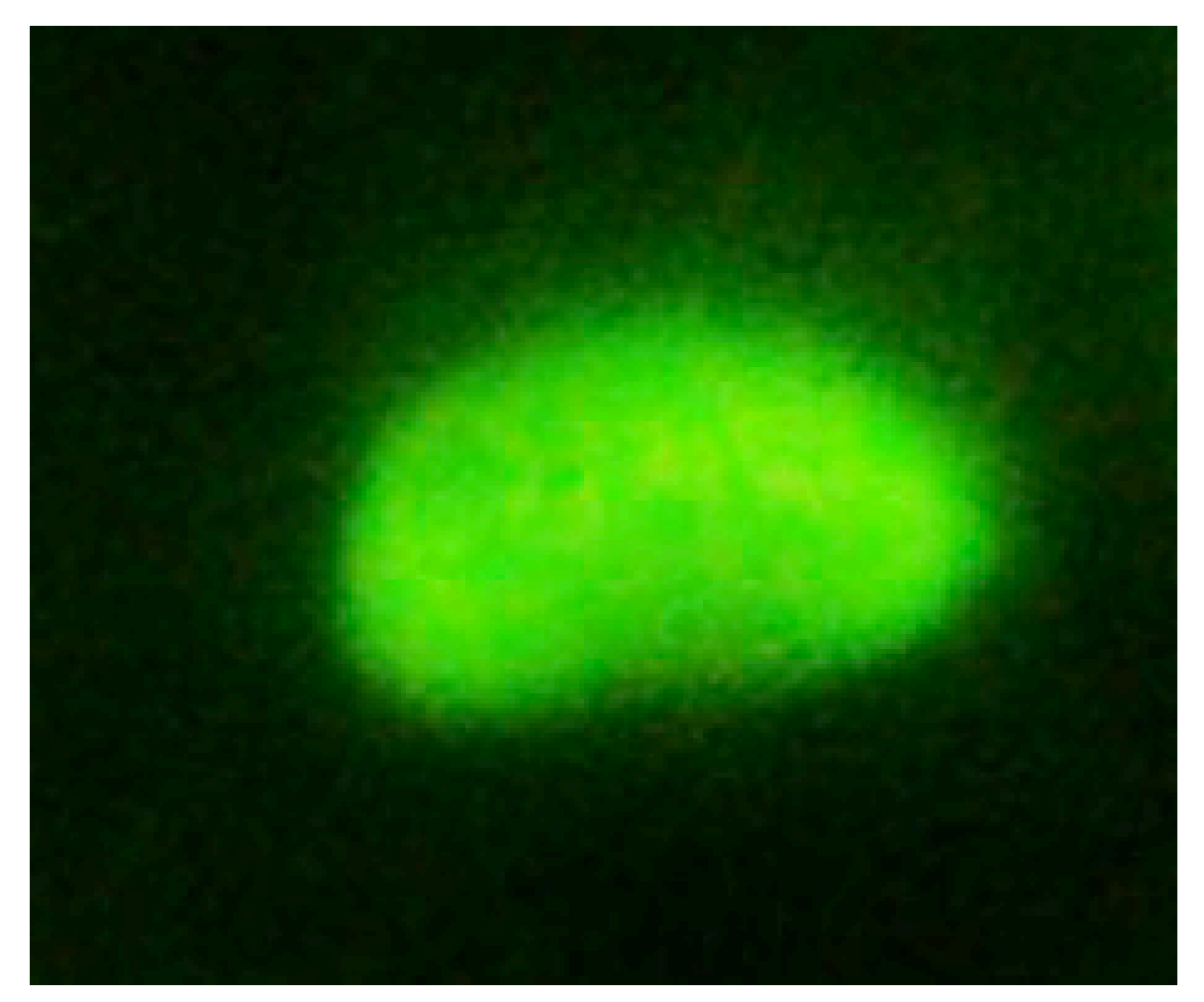	ellipsoidal	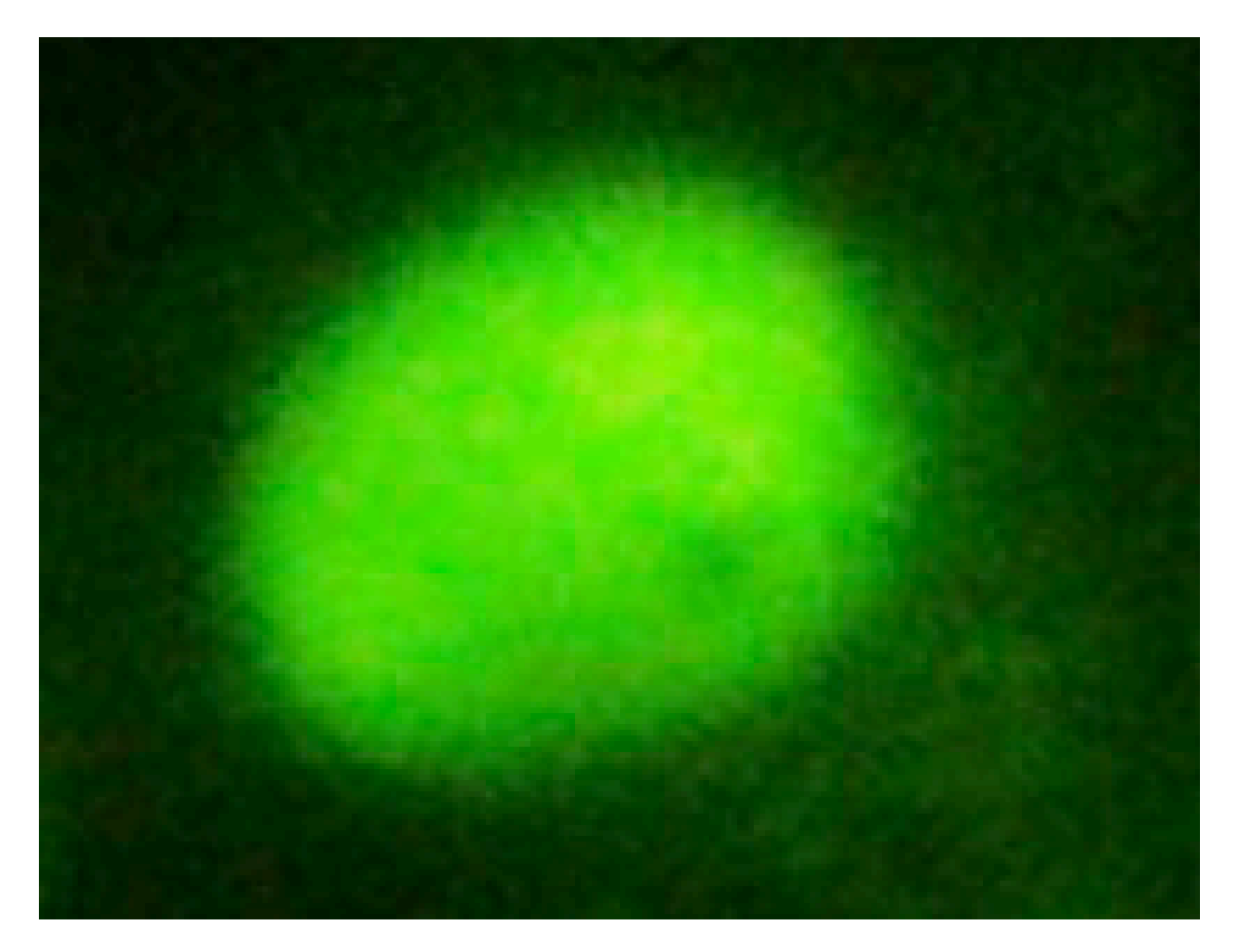	
Lemma	circular (central region)	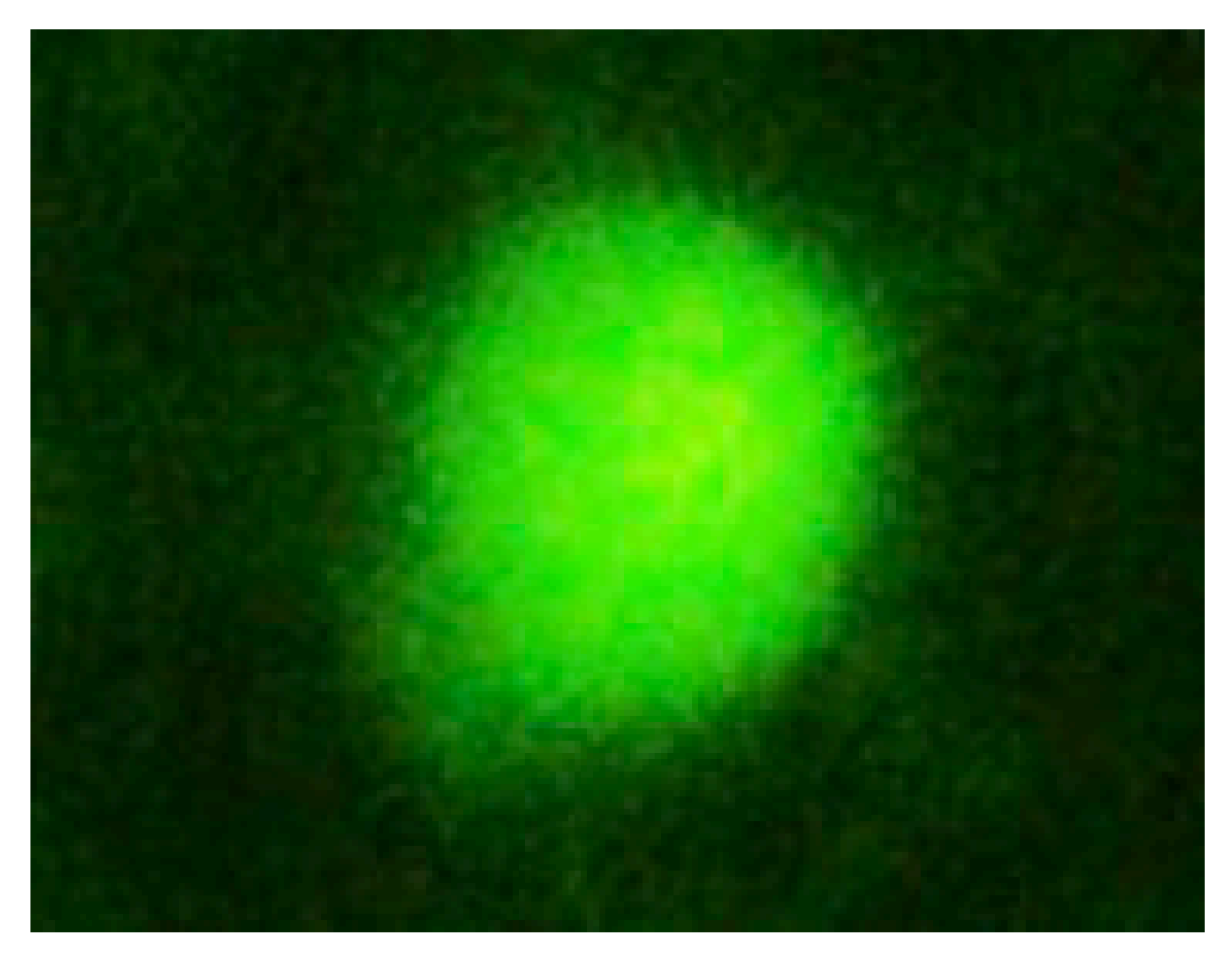	reniform ellipsoidal (margin)	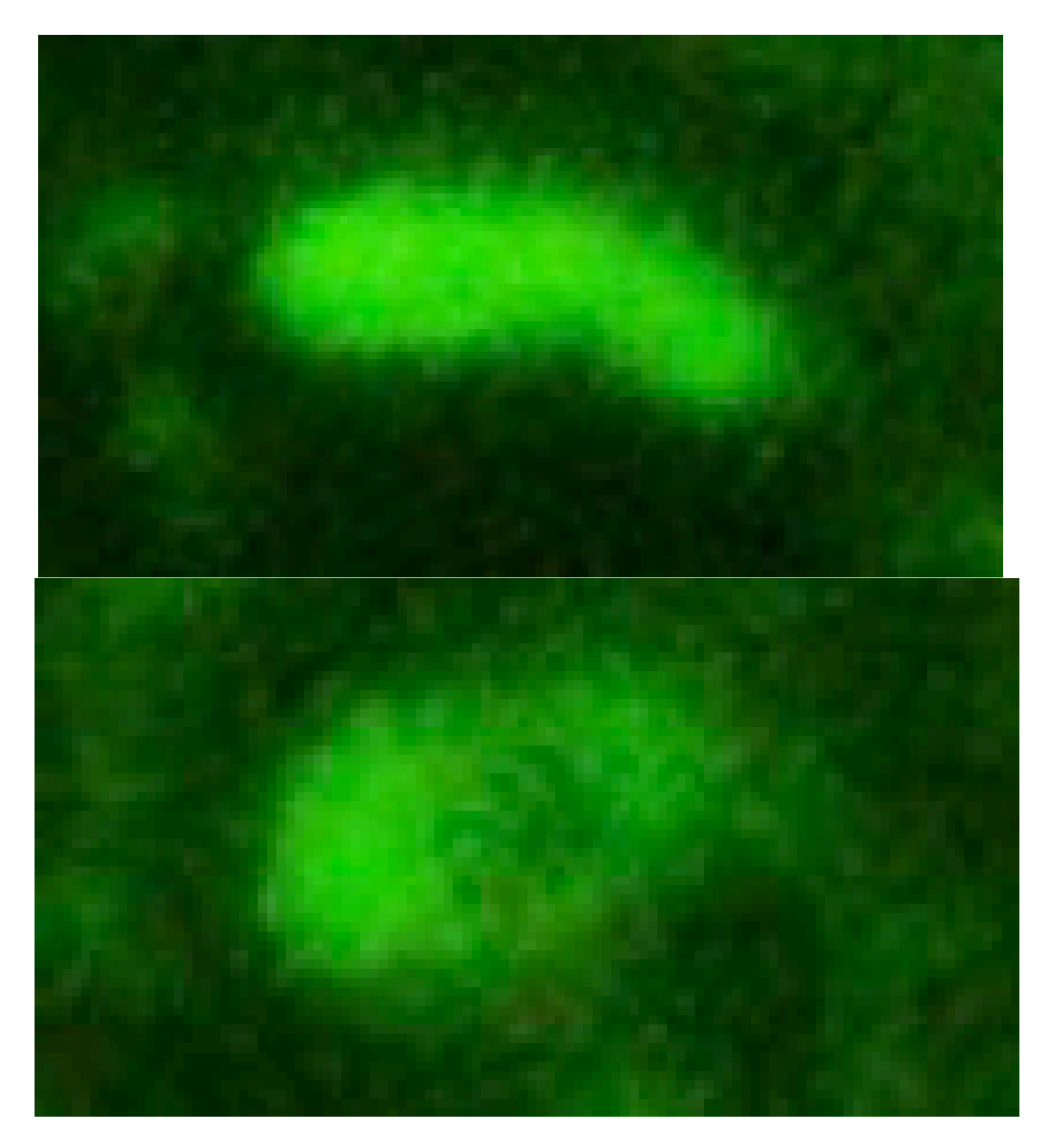	reniform and ellipsoidal silica bodies are small
Palea	barrel (central region)	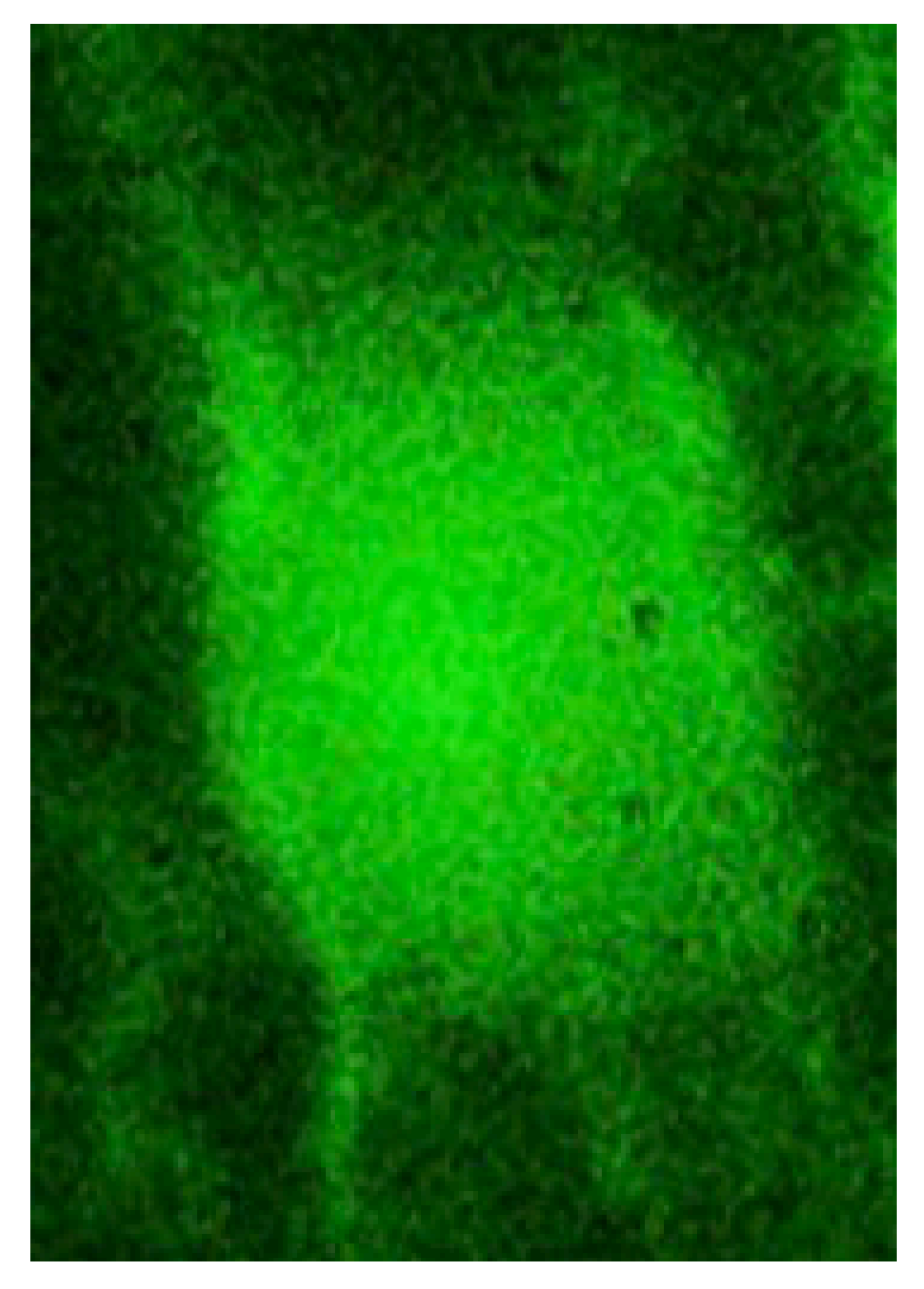	polylobate reniform ellipsoidal (margin)	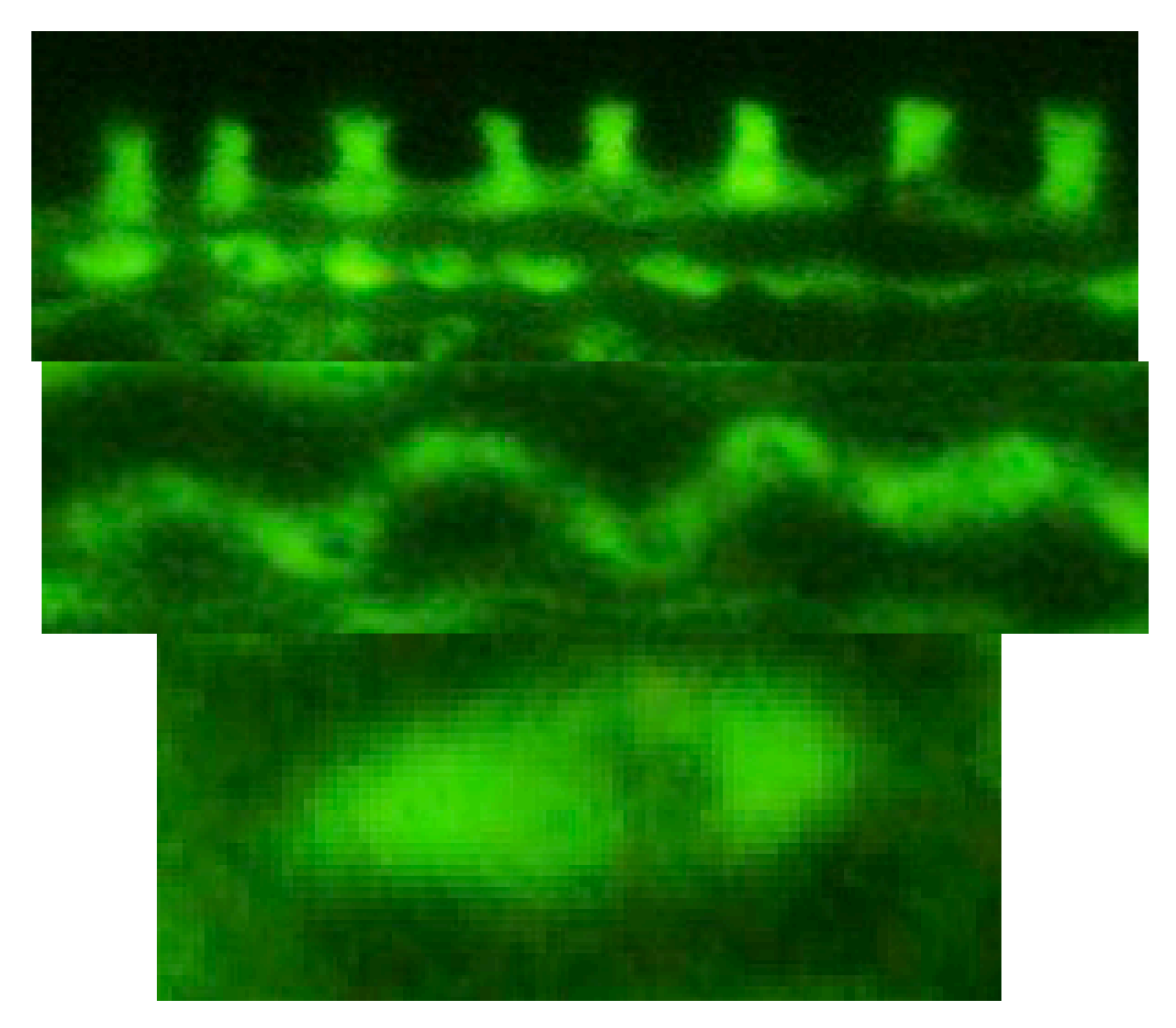	reniform and ellipsoidal silica bodies are small

**Table 2:** Silica-body size distributions in mature soil-grown controls and *in vitro*-grown plants following transfer to soil. L2D7 and L2D14, Leaf 2 sampled at Days 7 and 14 after transfer, respectively; L3D0, fully expanded Leaf 3 at Day 0 (negative control); L3D7 and L3D14, Leaf 3 sampled at Days 7 and 14 after transfer, respectively. Values are pooled descriptive measurements; individual silica bodies are nested within plants.

Group	Independent Plants (n)	Total Silica Bodies Measured (n)	Mean ± SD (µm^2^)	Median (µm^2^)	Range (µm^2^)
Mature soil-grown control (positive control)	5	259	88.9 ± 30.8	87	36–146
L2D7	12	376	65.3 ± 32.1	58	29–143
L2D14	12	391	72.9 ± 27.1	64	37–143
L3D0 (Day 0 negative control)	12	0	0	0	0
L3D7	12	381	72.2 ± 26.2	69	31–144
L3D14	12	386	77.0 ± 25.6	71	34–146

## Data Availability

The original contributions presented in this study are included in the article/Supplementary Materials. Further inquiries can be directed to the corresponding author.

## Supplementary Materials

The following supporting information can be downloaded at: https://www.mdpi.com/article/10.3390/plants15213476/s1, Figure S1: Relatively uniform spatial patterns of silica body deposition in leaf blades during post-transplant silicification. Samples were collected at 7 and 14 days after transfer from MS medium to soil, and silicification was examined in Leaf 2 (L2) and Leaf 3 (L3). (A,C) L2 at Days 7 and 14, respectively; (B,D) higher-magnification views of the boxed regions in A and C; (E,G) L3 at Days 7 and 14, respectively; and (F,H) higher-magnification views of the boxed regions in (E,G). Panels (A,C,E,G) are shown at the same magnification (scale bar = 100 µm); panels (B,D,F,H) are shown at the same magnification (scale bar = 10 µm).
